# Analysis of TRPV channel activation by stimulation of FCεRI and MRGPR receptors in mouse peritoneal mast cells

**DOI:** 10.1371/journal.pone.0171366

**Published:** 2017-02-03

**Authors:** A. Solís-López, U. Kriebs, A. Marx, S. Mannebach, W. B. Liedtke, M. J. Caterina, M. Freichel, V. V. Tsvilovskyy

**Affiliations:** 1 Pharmakologisches Institut, Ruprecht-Karls-Universität Heidelberg, Heidelberg, Baden-Württemberg, Germany; 2 Experimentelle und Klinische Pharmakologie und Toxikologie, Universität des Saarlandes, Homburg, Saarland, Germany; 3 Department of Neurology, School of Medicine Duke University, Durham, North Carolina, United States of America; 4 Departments of Neurosurgery, Biological Chemistry, and Neuroscience, Neurosurgery Pain Research Institute, The Johns Hopkins School of Medicine, Baltimore, Maryland, United States of America; Indiana University School of Medicine, UNITED STATES

## Abstract

The activation of mast cells (MC) is part of the innate and adaptive immune responses and depends on Ca^2+^ entry across the plasma membrane, leading to the release of preformed inflammatory mediators by degranulation or by *de novo* synthesis. The calcium conducting channels of the TRPV family, known by their thermo and osmotic sensitivity, have been proposed to be involved in the MC activation in murine, rat, and human mast cell models. So far, immortalized mast cell lines and nonspecific TRPV blockers have been employed to characterize the role of TRPV channels in MC. The aim of this work was to elucidate the physiological role of TRPV channels by using primary peritoneal mast cells (PMCs), a model of connective tissue type mast cells. Our RT-PCR and NanoString analysis identified the expression of TRPV1, TRPV2, and TRPV4 channels in PMCs. For determination of the functional role of the expressed TRPV channels we performed measurements of intracellular free Ca^2+^ concentrations and beta-hexosaminidase release in PMCs obtained from wild type and mice deficient for corresponding TRPV1, TRPV2 and TRPV4 in response to various receptor-mediated and physical stimuli. Furthermore, substances known as activators of corresponding TRPV-channels were also tested using these assays. Our results demonstrate that TRPV1, TRPV2, and TRPV4 do not participate in activation pathways triggered by activation of the high-affinity receptors for IgE (FcεRI), Mrgprb2 receptor, or Endothelin-1 receptor nor by heat or osmotic stimulation in mouse PMCs.

## Introduction

Mast cells (MC) mediate an optimal host defense against bacterial and parasitic infections [1, 2], but also pathology associated disorders, such as allergic and autoimmune disorders [3, 4]. It is well known that a sustained elevation of intracellular Ca^2+^ concentration ([Ca^2+^]_i_) is a mandatory signal for mast cell activation and degranulation processes induced by multiple stimuli, including immunological inputs, basic secretagogues, and physical cues [5, 6]. The best studied mechanism of the immunological activation of mast cells involves the aggregation of the high-affinity receptors for IgE (FcεRI). FcεRI- mediated Ca^2+^ entry is largely reduced in bone marrow derived mast cell cultures lacking ORAI1 proteins [7]. However, these cells still exhibit a significant residual Ca^2+^ entry following FcεRI stimulation, suggesting the participation of other ion channels, possibly other members of the Orai family and/or TRP channels. Furthermore, mast cells promote homeostasis by limiting the toxicity associated with endogenous mediators. For example, it has been described that mast cells modulate endothelin-1 (ET-1) levels to prevent pathological conditions. ET-1 binds to the endothelin-a receptor (ET_A_), which leads to the activation and degranulation of mast cells. Finally, the released proteases degrade ET-1 [8]. It has been shown that ET-1 induces an increase in [Ca^2+^]_i_ [9]. However, the molecular constituents of the calcium channels that mediates this [Ca^2+^]_i_ rise have not been identified. An additional property of mast cells is their IgE-independent pathway responsiveness to basic secretagogues, which trigger an [Ca^2+^]_i_ rise and degranulation in mast cells by activating the G-protein-coupled receptor Mrgprb2 via numerous basic secretagogues including compound 48–80 (48–80) [10]. Nevertheless, up to now it is not known which calcium channel is involved in the [Ca^2+^]_i_ elevation triggered by Mrgprb2 receptor. It has been described that mast cells have a direct responsiveness to physical stimuli, such as thermal, osmotic, and mechanical perturbations. In fact, it is known that inappropriate activation of mast cells by a brief exposure to heat, pressure, or light can cause urticarias [11–13]. Recent studies show the participation of epidermal TRPV4 channels in modulation of pain sensitivity in the skin in response to UVB over-exposure, where primary sensory neurons that innervate UVB-overexposed skin receive pro-pain signals from epidermal keratinocytes via an autocrine/paracrine feed-forward loop that involves increased ET-1 expression/secretion and activation of TRPV4 channels expressed in keratinocytes [14]. Specifically, Ca^2+^-entry in keratinocytes as well as pruritus evoked by ET-1 as well as by 48–80 is largely reduced in mice with genetically-encoded Trpv4 deficiency in keratinocytes, with inducible deletion of Trpv4 [15]. HoweveroHow, the contribution of TRPV4 in mast cells was not analyzed so far. Furthermore, it has been published that heat triggers [Ca^2+^]_i_ elevation and degranulation in HMC-1 cells, a human mast cell line [12].

Physical stimuli have been shown to be mediated through mechano- or thermosensitive transient receptor potential channels of the vanilloid subfamily (TRPV). All members of the TRPV subfamily, except TRPV3 and TRPV5, were reported to be expressed in various mast cells models [6]. For instance, the transcripts of TRPV1, TRPV2, TRPV4, and TRPV6 were identified in RBL-2H3 cells (rat basophilic leukemia cell line) [11, 16], TRPV1, TRPV2, and TRPV6 in HMC-1 [12, 17], and TRPV2 in human lung, skin, and cord blood-derived mast cells [18]. TRPV1 proteins were detected in mast cells obtained from the human bladder [19] and skin [20]. Nonetheless, most of the experiments testing the role of TRPV channels in mast cells have been carried out in transformed cells lines such as HMC-1 which grow independent of the stem cell factor (SCF) and might have a limited functional significance [21]. As primary mast cell models, bone marrow-derived mast cells (BMMCs) and mast cells cultured from the peritoneal lavage (PMCs) are most commonly used. PMCs represent a valuable mast cell model that resembles connective tissue type mast cells, which predominate e.g. in the skin and are activated during the development of cutaneous anaphylaxis [5, 22]. Furthermore, except for TRPV1, antagonists of the TRPV family members have insufficient potency or specificity. Therefore, studies to reveal their function and activation mode in primary cells still rely on experiments using genetic approaches and transgenic animals. We were interested to investigate the functional relevance of individual TRPV proteins in their native environment for cation influx into primary isolated cells systems. To this end, we used PMCs isolated from mouse models deficient for TRPVs channels and evaluate their role in the signaling pathway triggered by multiple stimuli. Our results indicate that TRPV1, TRPV2, and TRPV4 do not participate in the mouse PMCs activation pathways triggered by immunological inputs mediated by FcεRI stimulation, ET_A_, basic secretagogues mediated by Mrgprb2, and physical cues, such as heat and osmotic stress.

## Materials and methods

### Solutions and chemicals

Fura-2 AM and RPMI 1640 media was from Thermo Fisher Scientific. The rest of the compounds were purchased from Sigma-Aldrich Chemical Co. Physiological Solution (PSS) in mM: 135 NaCl, 6 KCl, 2 CaCl_2_, 1,2 MgCl_2_, 10 HEPES, 12 D-glucose, 310 mOsm and pH = 7.4 with NaOH. Tyrode Solution (TS) in mM: 130 NaCl, 5 KCl, 2 CaCl_2_, 1 MgCl_2_, 10 HEPES, 5,6 D-glucose, 0.01% BSA, 300 mOsm and pH = 7.4 with NaOH.

### Animals

Mice were obtained from the Animal Facility (Interfakultäre Biomedizinische Forschungseinrichtung) of University of Heidelberg and were housed under standard conditions and supplied with drinking water and food ad libitum. Mice were euthanized by CO_2_ exposure followed by cervical dislocation. All animal procedures fulfilled the German legislation guidelines for care and use of laboratory animals (officially approved by the Karlsruhe regional council). The generation of the TRPV1 and TRPV2 deficient mice was described by Caterina MJ. et al. [23] and Park U. et al. [24], respectively. The generation of the TRPV4 deficient mice was described by Liedtke et al. [25]. The genetic background of the TRPV1, TRPV2, and TRPV4 mouse line is C57Bl6/N, 129S6, and C57Bl6/J, respectively. The appropriate WT controls were always used for each independent preparation of PMCs.

### Peritoneal mast cell isolation and cultivation

PMCs were collected through a lavage of the abdomen of the mice according to Ryazanova et al. [26]. Briefly, RPMI medium was injected into the peritoneum of dead mice and incubated with agitation during 60 seconds. The medium with the cells is aspirated, centrifuged, and resuspended in RPMI medium with 20% FBS, 1% Penicillin/Streotomycin, IL-3 (10 ng/ml), and SCF (30 ng/ml). The cells (1x10^6^/ml) were cultured between 14 to 16 days at 37°C and 5% CO_2_ before the experiments were conducted. To measure the [Ca^2+^]_i_ rise or degranulation mediated by FcεRI, we sensitized PMCs by incubating them with 300 ng/ml αDNP-IgE overnight (anti- dinitrophenyl antibody IgE isotype), followed by DNP (2,4-Dinitrophenyl hapten conjugated to Human Serum Albumin protein) exposure.

### Nanostring nCounter and RT-PCR expression analysis

Total RNA from mouse PMCs was isolated using the Qia RNeasy Mini Kit (Qiagen, Germany) following the instructions of the manufacturer. Quantity, purity, and integrity of the RNA samples were controlled by spectrophotometry (NanoQuant, Tecan) and microfluidic analysis (Bioanalyzer 2100, Agilent Technologies). The NCounter NanoString technology allows digital quantifying of transcripts in three steps as described by Geiss GK. et al. [27]. Briefly, in the first step two probes (the reporter and the capture probe) hybridize directly to the target molecule in solution. Then, the target-probe complexes are immobilized in the imaging surface of the nCounter Cartridge by binding of the capture probe. Finally, the sample cartridges are scanned by an automated fluorescence microscope and molecular barcodes (fluorophores contained in the reporter probe) for each specific target are counted. For expression analysis by NCounter NanoString technology, 1 μg total RNA was hybridized (biological replicates, RIN> 8.3) with a Nanostring Gene Expression CodeSet and analysed using the nCounter Digital Analyzer. Background correction was performed and normalization was applied using 5 different housekeeping genes (Hprt1, Tbp, Ubc, Gapdh, Actb). Following Trpv-specific DNA sequences were used: Trpv1: 5’-GAGAAGATGATCCTCAGAGACCTGTGTCGGTTTATGTTCGTCTACCTCGTGTTCTTGTTTGGATTTTCCACAGCCGTAGTGACACTGATCGAGGATGGGA-3’, Trpv2: 5’-GAACCTGCTTTATTATACACGTGGCTTTCAGCACACAGGCATCTACA GTGTCATGATCCAAAAGGTCATTCTGCGAGACCTGCTCCGCTTCCTGCTGGT-3’, Trpv3: 5’-ATGTTTGTCCTCATCTGGGCCACATGCATCTCTGTGAAAGAAGGC ATTGCCATTTTCCTGCTGAGACCCTCCGATCTTCAGTCCATCCTGTCAGATGCCT-3’, Trpv4: 5’-GGCTGGCTGGCGAGGTCATCACGCTCTTCACAGGAGTCC TGTTCTTCTTTACCAGTATCAAAGACTTGTTCACGAAGAAATGCCCTGGAGTGAATTCTCT-3’, Trpv5: 5’-CTGTGGTTGCGAATACGGCCTGGGAGACCGTTGGT TCTTACGGGTTGAACACCACCAGGAGCAGAATCCTTATCGAGTACTTCGATATGTGGAAGCTTTC-3’,Trpv6: 5’-CCAGAACTAGTCTTTGAGCCCATGACTTCGGA GCTATATGAAGGTCAGACTGCACTACACATTGCAGTAATAAACCAGAATGTAAACTTGGTCCGTGCTC-3’.

Total RNA (100 ng) isolated from PMCs was used for one step reverse transcription-PCR (RT-PCR, Invitrogen). The following intron-spanning primers were used for amplification of TRPV1, TRPV2, TRPV3, TRPV4, TRPV5, and TRPV6 fragments, respectively: TRPV1: 5’- GCA TCT TCT ACT TCA ACT TCT TCG TC -3’ and 5’- CCA CAT ACT CCT TGC GAT GGC -3’ resulting in a 320 bp fragment; TRPV2: 5’- CGG CAC TTC CTC TCT -3’ and 5’- GTC GGT CAC GGT CAA—3’ resulting in a 427 bp fragment; TRPV3: 5’- CAA GGA CTG CCA CCA CCA TC -3’ and 5’- CAT CAC AGT TGC CAG AGA GG—3’ resulting in a 298 bp fragment; TRPV4: 5’- GAG TCC TCA GTA GTG CCT GG -3’ and 5’- CAA CAA GAA GGA GAG CAG TC -3’ resulting in a 249 bp fragment; TRPV5: 5’- CAG TGA AGG AGC TGG TGA GC -3’ and 5’- CCA GCC GGA TCT TAT CCT GG -3’ resulting in a 225 bp fragment; TRPV6: 5’- CCT CGT TGC CTG TGG CCT C-3’ and 5’- CAC CAA AGG GAC GTG CTG TC -3’ resulting in a 208 bp fragment. In control experiments, total RNA from mouse testis, brain, tongue, kidney, or placenta isolated using PeqGold (PeqLab, Germany) was used.

### Fluorometric analysis of Ca^2+^ signaling

For the fluorometric analysis we used Fura-2, which is a ratiometric Ca^2+^ sensitive fluorescent dye. Cells were loaded with 2 μM Fura-2 AM during 30 min on coverslips pretreated with 0.01% Poly-L-Lysine for at least 30 min (to attach the cells to the glass). We measured the time course of [Ca^2+^]_i_ in cells in PSS and at room temperature in a single cell imaging setup composed of an inverted Zeiss Axio Vert.A1 microscope equipped with a 40X Fluor/1.3 NA objective. Fluorescent illumination was provided by a monochromator (Polychrome V, Till Photonics). The cells were excited alternatively at 340 and 380 nm during 50 ms and the emission light of wavelengths higher than 510 nm was collected. F340/380 ratio was collected every 5 s using an ORCA-Flash4.0 V2 Digital CMOS Camera (Hamamatsu Photonics) operated with the ZEN 2 pro software (Zeiss). At least 200 cells were recorded for each independent preparation. For the thermal stimulation, the cells were perfused with preheated PSS. The temperature solution was regulated with the temperature control system TC-20 (NPI Electronic).

### β-hexosaminidase degranulation assay

The β-hexosaminidase enzyme was quantified by spectrophotometric analysis of 4-Nitrophenyl N-acetyl-β-D-glucosaminide (pNAG) hydrolysis as previously described [28]. Briefly, PMC were suspended in TS and seeded in a 96-well plate (1x10^5^ cells / well). Degranulation was induced by incubating PMC in the presence of the agonist during 45 min at 37°C and 5% CO_2_. For the thermal stimulation, the cells were incubated in a thermomixer (Eppendorf) at 52°C during 5 up to 30 min. Cells are centrifuged at 1,200 r.p.m. for 5 min at 4°C. Cell lysates (solubilized with 1% Triton-X during 5 min) and supernatants were incubated separately with 4mM pNAG for 1 h at 37°C and the reaction was stopped with 200mM glycine (pH 10.7). A dual-wavelength was used at 405 and at 630 nm and the absorbance was quantified with the NanoQuant infinite M200pro (Tecan, Switzerland). In order to eliminate the background noise, the absorbance value obtained at 630 nm was subtracted to the value obtained at 405 nm. The degranulation assay was always performed in duplicates for each independent preparation. The percentage of β-hexosaminidase release was calculated according to following equation % βHex = (S/(S + C))x100, where S and C are the supernatant and the cell lysate, respectively.

### Data analysis

Data analysis was made with Origin 8.5 (OriginLab Corporation, USA) or Prism 5.0c (GraphPad Software Inc.) software. The results are presented as mean ± SEM of n independent experiments. All analyses were made using each independent PMC preparation as an experimental unit. Statistical analyses were performed using the appropriate tests (unpaired doubled tailed t-test or one-way ANOVA).

## Results and discussion

**Fig 1:**
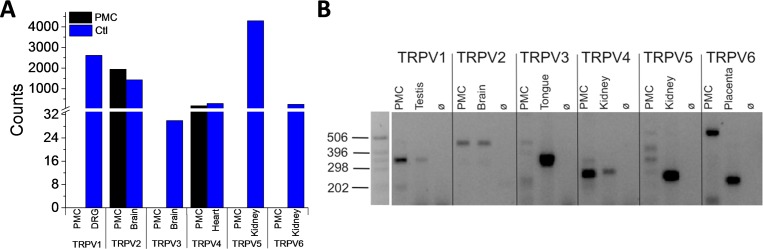
TRPV1, TRPV2, and TRPV4 are expressed in peritoneal mast cells. Total RNA from mouse PMCs was isolated. The nCounter NanoString system (A) and RT-PCR technique (B) were employed for expression analysis. NanoString analysis shows the number of transcripts (Counts) per analyzed gene. As positive control, neurons from the dorsal root ganglion (DRG) and tissue from the indicated organs were used In the RT-PCR experiments, RNA extracts without reverse transcriptase served as negative controls (ø). At least 3 independent PMCs preparations were analyzed.

In order to identify expression in PMCs of the individual members of the TRPV subfamily, we first employed the nCounter NanoString technology. As indicated in Fig 1A, we detected transcripts of TRPV2 and TRPV4. In addition, we performed reverse transcription PCR, which is a more sensitive technique to detect transcripts. The results confirmed the TRPV2 and TRPV4 expression and also indicated expression of TRPV1 (Fig 1B). However, no amplification products of the expected size were detected for TRPV3, 5, or 6 using this approach.

**Fig 2:**
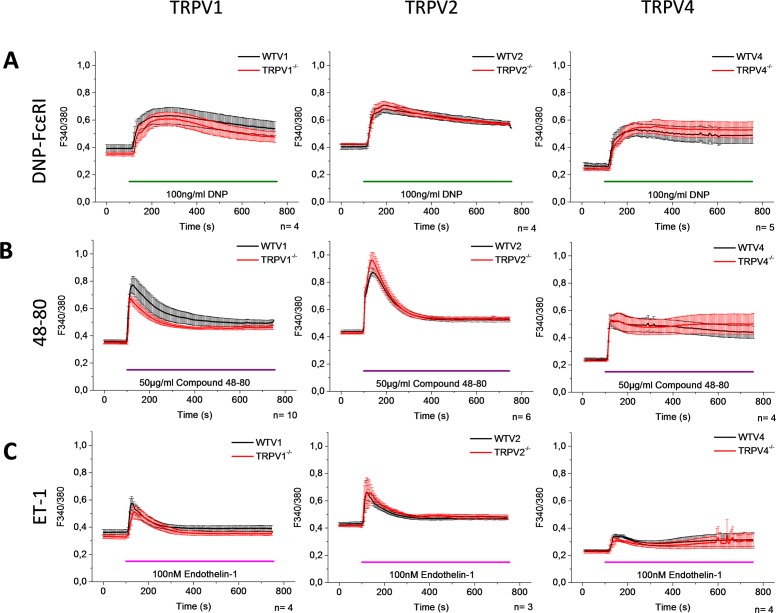
TRPV1, TRPV2, and TRPV4 do not participate in the elevation of [Ca^2+^]_i_ triggered by DNP-FcεRI, Compound 48–80, and Endothelin-1. PMCs were obtained from WT mice (black) and mice deficient for TRPV1, TRPV2, and TRPV4 (red). Graphs show the time course of [Ca^2+^]_i_ changes in Fura-2 loaded PMCs stimulated with 100ng/ml DNP-FcεRI^**ᵻ**^ (A), 50μg/ml Compound 48–80 (B), and 100nM Endothelin-1 (C). Data are presented as the 340/380-nm fluorescence ratio (F340/380). ^**ᵻ**^Cells were preincubated with αDNP-IgE overnight. Graphs represent the mean ± SEM of at least 3 independent PMCs preparations.

**Fig 3:**
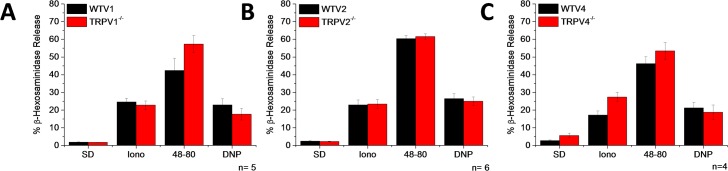
TRPV1, TRPV2, and TRPV4 do not participate in the degranulation process triggered by DNP-FcεRI or Compound 48–80. PMCs were obtained from WT mice (black) and mice deficient (red) for TRPV1 (A), TRPV2 (B), or TRPV4 (C). Bar graphs show the percentage of β-hexosaminidase release. SD-Spontaneous degranulation, Iono- 10μM Ionomycin, 48-80- 50μg/ml Compound 48–80, and 100 ng/ml DNP. ^**ᵻ**^Cells were preincubated with αDNP-IgE overnight. Graphs represent the mean ± SEM of at least 4 independent PMCs preparations.

To assess the function of TRPV1, TRPV2, and TRPV4 in the signaling pathway of the most commonly used mast cell activators, we measured [Ca^2+^]_i_ changes and degranulation in PMCs isolated from wild type (WT) and TRPV1, TRPV2, and TRPV4 deficient mice. For the [Ca^2+^]_i_ measurements, PMC were loaded with Fura2-AM and the [Ca^2+^]_i_ changes were recorded by single cell imaging. We evaluated the response mediated by FcεRI, Mrgprb2, and ET_A_ by stimulating the cells with DNP, Compound 48–80 (48–80), and ET-1, respectively. As shown in Fig 2, we did not observe any difference in the [Ca^2+^]_i_ rise triggered by these compounds in the PMCs isolated from the TRPV1, TRPV2, or TRPV4 deficient mice compared to the PMCs isolated from the corresponding WT controls, respectively. Nevertheless, this does not necessarily exclude that these channels can contribute in the degranulation process in PMC, since their contributions might only occur in specific nanodomains of the cells, such that a difference cannot be identified in the global [Ca^2+^]_i_ measurement, which has been shown to occur in other cell types [29]. To characterize the contribution of the TRPV channels in the overall degranulation process, we measured β-hexosaminidase release after exposure to DNP-FcεRI or 48–80. Spontaneous and ionomycin-induced degranulation measurements were used as a negative and positive control, respectively. Fig 3 shows that the percentage of degranulation triggered by DNP-FcεRI and 48–80 is not different between the PMCs that were isolated from the TRPV1, TRPV2, or TRPV4 deficient mice, compared to PMCs isolated from the corresponding WT controls. Altogether, these results indicate that TRPV1, TRPV2, and TRPV4 do not participate in either the [Ca^2+^]_i_ rise nor degranulation mediated by FcεRI, Mrgprb2, and ET_A_ receptors. Comparatively, ET-1 and 48–80 evoked Ca^2+^ entry was largely reduced in keratinocytes of TRPV4 deficient mice [15].

**Fig 4:**
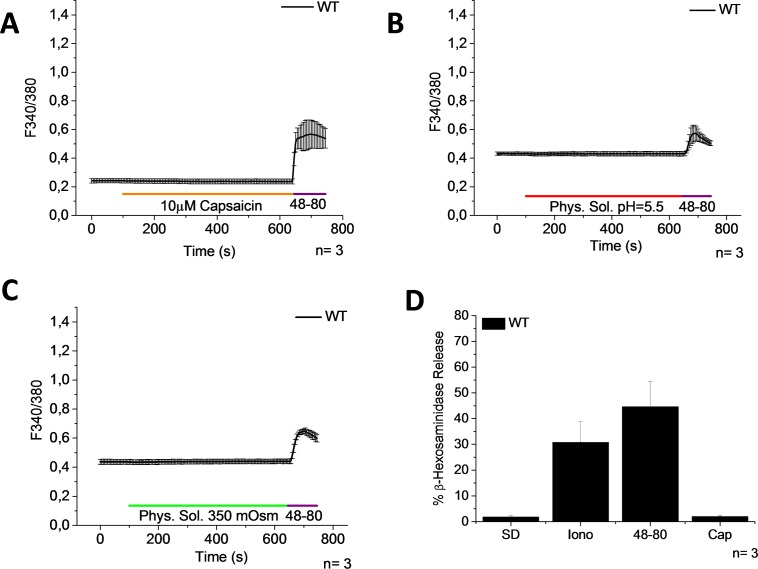
Activators of TRPV1 induce neither an increase of [Ca^2+^]_i_ nor degranulation. PMCs were obtained from WT mice. (A-C) Time course of [Ca^2+^]_i_ changes in Fura-2 loaded PMCs stimulated with 10μM Capsaicin (A), protons, pH = 5.5 (B), and hypertonic solution, +50 mOsm kg–1 (C). 1 μg/ml 48–80 was used as a positive control. Bar graph shows the percentage of β-hexosaminidase release. SD- Spontaneous degranulation, Iono- 10μM Ionomycin, 48-80- 50μg/ml Compound 48–80, and Cap- 10μM Capsaicin. Graphs represent the mean ± SEM of at least 3 independent preparations.

To measure directly whether TRPV channels are functionally active in PMCs, we exposed the cells to specific stimulators of TRPV1, TRPV2, and TRPV4 channels that were reported in different cell models [30]. We stimulated the cells with capsaicin, the most commonly used activator of TRPV1. However, neither a [Ca^2+^]_i_ rise nor degranulation was induced by capsaicin (Fig 4A and 4D). The activity of the capsaicin used was confirmed by measuring a [Ca^2+^]_i_ rise in HEK 293 cells that constitutively express TRPV1 (S1 Fig). Our results are in accordance to previous reports, where it was shown that capsaicin induces Ca^2+^ uptake in BMMCs but not in PMCs [13]. It is well known that TRPV1 responds to protons [31] and to hypertonic stimuli [32]. Consequently we perfused the cells with a physiological solution with a pH = 5.5 and a hypertonic solution (+50 mOsm kg–1). However, a [Ca^2+^]_i_ rise was not observed in PMCs with either of these stimuli (Fig 4B and 4C). Taken together, our results suggest that the TRPV1 channel is not functional in PMCs.

**Fig 5:**
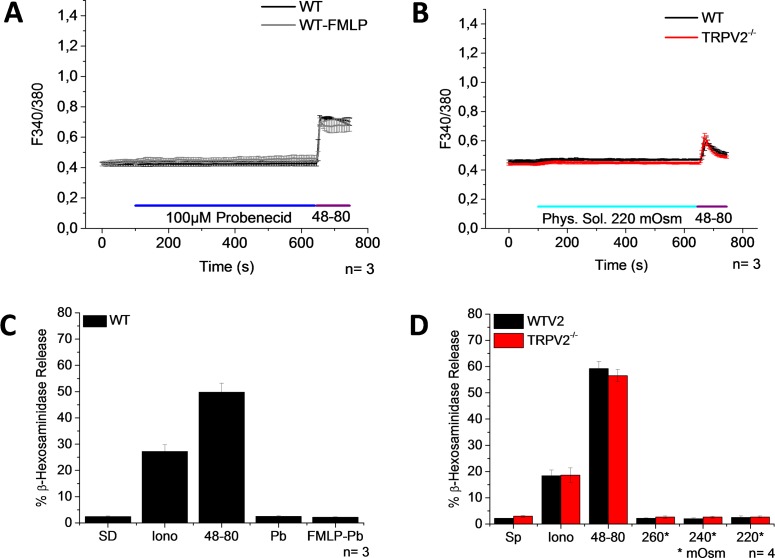
Activators of TRPV2 induce neither an increase of [Ca^2+^]_i_ nor degranulation. PMCs were obtained from WT mice. (A-B) Time course of [Ca^2+^]_i_ changes in Fura-2 loaded PMCs stimulated with 100μM Probenecid in the presence or absence of 30μM FMLP (A) or with hypotonic solution, -90 mOsm kg–1 (B). 1 μg/ml 48–80 was used as a positive control. (C-D) Bar graphs show the percentage of β-hexosaminidase release. SD-Spontaneous degranulation, Iono- 10μM Ionomycin, 48-80- 50μg/ml Compound 48–80, and Pb- 100μM Probenecid, 30μM FMLP (C), and hypotonic solution, 280, 240, and 220 mOsm kg–1 (D). Graphs represent the mean ± SEM of at least 3 independent preparations.

It has been shown that probenecid is a specific TRPV2 activator within TRPV channels [33] and that in macrophages, FMLP induces the translocation of TRPV2 from intracellular compartments to the plasma membrane [34]. For that reason, we stimulated PMCs with probenecid with and without FMLP pretreatment. Nevertheless, neither a [Ca^2+^]_i_ rise nor degranulation was detected (Fig 5A and 5C). It has been shown that TRPV2 activity is modulated by hypotonic stress [35]. However, we did not observe any [Ca^2+^]_i_ change following the application of a hypotonic solution to PMCs isolated from WT mice and mice deficient for TRPV2 (Fig 5B and 5D). The ability of probenecid and the hypotonic solution to active TRPV2 was confirmed in HEK293 cells that were transfected with a plasmid containing mTRPV2-IRES-GFP construct (S2 Fig).

**Fig 6:**
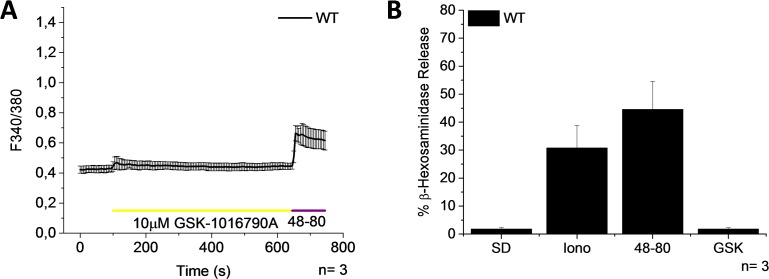
Activator of TRPV4 induces neither an increase of [Ca^2+^]_i_ nor degranulation. PMCs were obtained from WT mice. (A) Time course of [Ca^2+^]_i_ changes in Fura-2 loaded PMCs stimulated with 10μM GSK-1016790A. 1 μg/ml 48–80 was used as a positive control. (B) Bar graph shows the percentage of β-hexosaminidase release. SD-Spontaneous degranulation, Iono- 10μM Ionomycin, 48-80- 50μg/ml Compound 48–80, and GSK- 10μM GSK-1016790A. Graphs represent the mean ± SEM of at least 3 independent preparations.

To measure TRPV4 activity we exposed PMCs to 10 μM GSK-1016790A, a TRPV4 activator. Nonetheless, neither a [Ca^2+^]_i_ elevation nor degranulation was observed (Fig 6) in PMCs, indicating that the TRPV4 channel is not functional in PMCs. In contrast, 10 μM GSK-1016790A induces a [Ca^2+^]_i_ rise in cardiac fibroblasts extracted from WT mice, but not from TRPV4 deficient mice (S3 Fig), demonstrating that the GSK-1016790A used for the experiments is active and specific to TRPV4.

**Fig 7:**
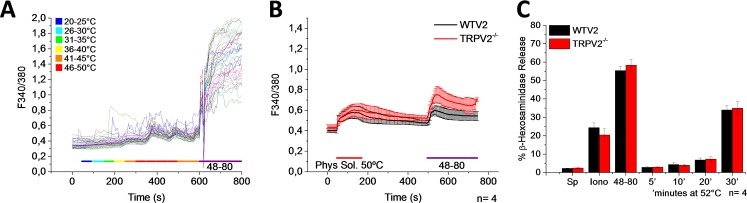
Heat triggers an increase of [Ca^2+^]_i_ and degranulation independently of TRPV2. PMCs were obtained from WT mice and mice deficient for TRPV2 (red). (A-B) Time course of [Ca^2+^]_i_ changes in Fura-2 loaded PMCs stimulated with heat. Representative single cell traces of [Ca^2+^]_i_ changes in response to a temperature ramp (from 20–50°C). Mean values of [Ca^2+^]_i_ elevation triggered by heat (50°C). 1 μg/ml 48–80 was used as a positive control. (C) Bar graph shows the percentage of β-hexosaminidase release. SD-Spontaneous degranulation, Iono- 10μM Ionomycin, 48-80- 50μg/ml Compound 48–80, and heat (incubation at 52°C for 5–30 minutes). Graphs represent the mean ± SEM of at least 3 independent preparations. Temperature color code in legend corresponds to colors in horizontal bar, but not to those in cell traces.

It is well known that several members of the subfamily of TRPV channels are activated by heat. The temperatures required to activate TRPV1, TRPV2, and TRPV4 channels are 42°C, 50°C, and 27°C, respectively [36–38]. Furthermore, it has been reported that in the human mast-cell line HMC-1 heat (bath solution at >50°C) induces a [Ca^2+^]_i_ rise and degranulation. This process has been suggested to be mediated by TRPV2, since in the presence of ruthenium red and SKF96365 the heat activation of HMC-1 is inhibited [12]. In order determine whether PMCs are also activated by heat, we increased the bath solution temperature from 20 to 46°C. As shown in Fig 7A, we observed a [Ca^2+^]_i_ rise at temperatures exceeding 46°C, which suggested the possibility that TRPV2 channels could mediate this process. Next, we measured [Ca^2+^]_i_ changes and degranulation in PMCs isolated from WT and TRPV2-deficient mice. The results shown in Fig 7B indicate that TRPV2 does not participate in the global [Ca^2+^]_i_ elevation induced by heat, since the response was also observed in cells that lack TRPV2. Degranulation was only observed after 30 min exposure of PMCs to 52°C, and there was no significant difference in the percentage of degranulation obtained from the PMCs isolated from WT vs. TRPV2 deficient mice, discarding TRPV2 involvement in the degranulation process evoked by high temperatures.

Very recently, Mascarenhas et al. published interesting results concerning mast cell signaling in the facial-skin-restricted dermatitis condition, Rosacea[39]. Their cell-based experiments on primary human mast cells, using the TRPV4 inhibitor HC-067, and on human CD34+ cord-blood-cell differentiated mast cells, using CRISPR/Cas-mediated TRPV4 knockdown, suggest that TRPV4 plays a significant role in MRGPRX2-mediated mast cell degranulation. The apparently different conclusions of our studies, with respect to TRPV4, could reflect a human vs. mouse species difference, a difference in the regionality of the mast cell subtypes studied, and/or different mast cell culture conditions. For example, in the study by Mascarenhas et al., mast cells may have been in a specific physiological state that was permissive for TRPV4 activation during MRGPRX2-mediated degranulation. Given their potentially wide clinical-medical relevance, these results merit dedicated follow-up studies, although this could be challenging if the differences between our studies are, in fact, attributable to species differences.

## Conclusion

Altogether, our results indicate that TRPV1, TRPV2, and TRPV4 do not participate in the signaling pathways triggered by calcium dependent secretagogues, heat or osmotic stimuli in mouse peritoneal mast cells. Additionally, we observed neither an increase of calcium nor degranulation when the PMCs were exposed to known activators of these TRP channels. Functional roles for TRPV1, TRPV2, and TRPV4 have been reported in other mast cell models such as rat and human derived mast cell lines. This concept, elaborated using immortalized mast cell lines and pharmacological inhibitors of TRPV channels, should not be generalized to mast cells as a whole, since our experiments using a rigorous approach in TRPV1, TRPV2, and TRPV4 deficient mice unequivocally rule out these TRPV channels as mediators of Ca^2+^ elevation and cell degranulation triggered by the activation of FcεRI, ET_A_, or Mrgprb2 receptors, or by physical cues in murine PMCs, cells that are commonly used as a model for connective tissue type mast cells. However, we cannot yet exclude the possibility that these channels function in other primary mast cell populations.

## Supporting information

S1 Fig Activator of TRPV1 triggers an increase of [Ca^2+^]_i_ in HEK293 cells expressing TRPV1 constitutively. Time course of [Ca^2+^]_i_ changes in HEK293 cells that constitutively express TRPV1 stimulated with 1 μM Capsaicin. Graphs represent the mean ± SEM of 3 independent preparations.
(TIF)

S2 Fig Activators of TRPV2 trigger an increase of [Ca^2+^]_i_ in HEK293 cells expressing TRPV2. Time course of [Ca^2+^]_i_ changes triggered by application of 100 μM of Probenecid (A) and 220 mOsm/kg hypotonic solution (B) in HEK293 cells that were transfected with a mTRPV2–IRES-GFP plasmid (black traces). TRPV2 expression was proven by the presence of the green fluorescence protein signal. As negative control, non-tranfected HEK293 cells were used (red traces). Ionomycin 1μM was used as a positive control. Graphs represent the mean ± SEM of 10 cells in each case.
(TIF)

S3 Fig Activator of TRPV4 triggers an increase of [Ca^2+^]_i_ in cardiac fibroblasts isolated from WT, but not TRPV4-deficient mice. Time course of [Ca^2+^]_i_ changes in Cardiac fibroblasts (CF) stimulated with 10 μM GSK-1016790A. CF were isolated from WT (black) and TRPV4 deficient (red) mice using retro-Langendorff-perfusion as previously described [40]. Graphs represent the mean ±SEM of 3 independent preparations.
(TIF)
