# Supplementary figures and images for: Analysis of TRPV channel activation by stimulation of FCεRI and MRGPR receptors in mouse peritoneal mast cells
Source: PLoS One. 2017 Feb 3;12(2):e0171366. doi: 10.1371/journal.pone.0171366 (PMC5291405; doi:10.1371/journal.pone.0171366)

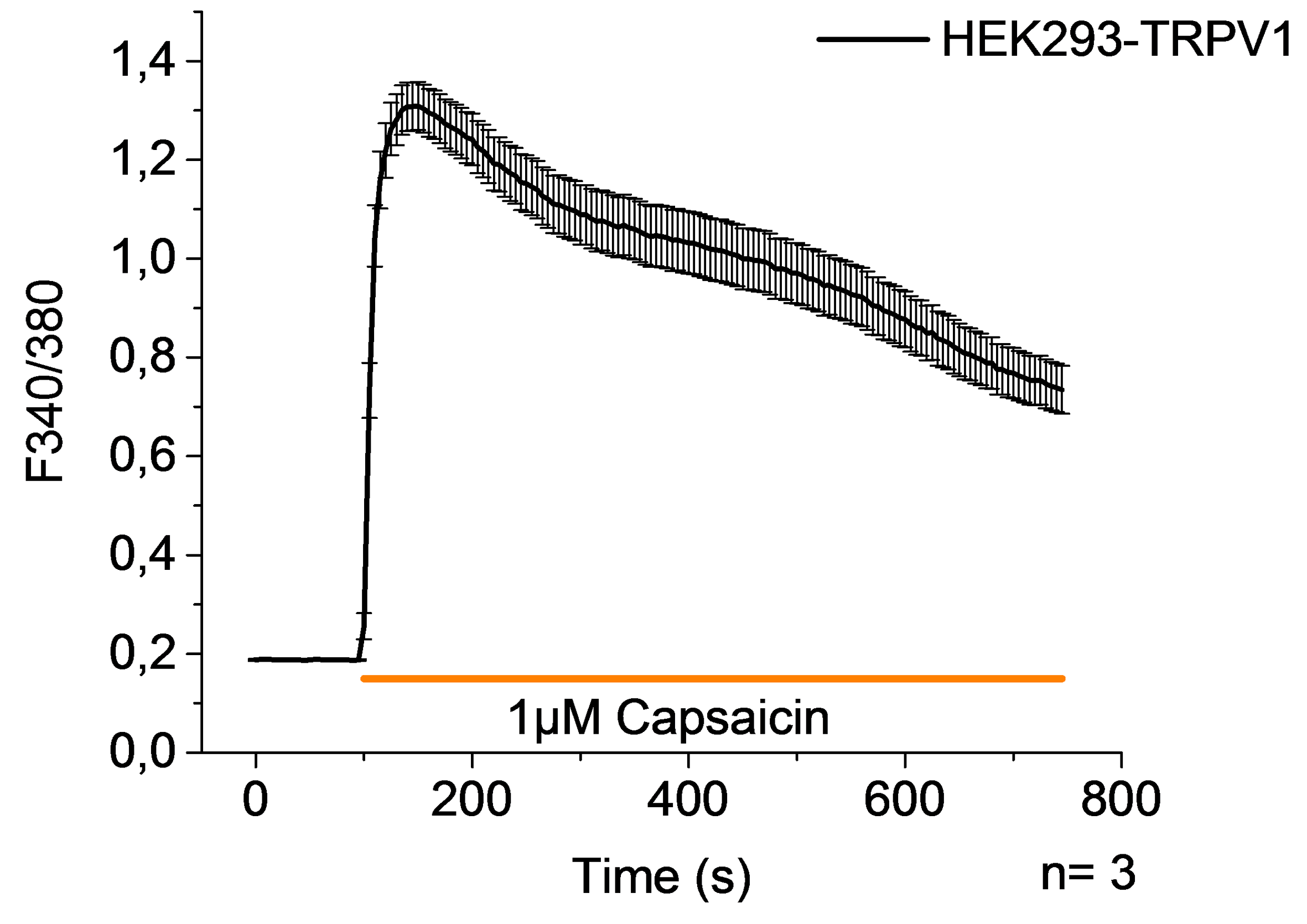

Supplement: S1 Fig — Time course of [Ca2+]i changes in HEK293 cells that constitutively express TRPV1 stimulated with 1 μM Capsaicin. Graphs represent the mean ± SEM of 3 independent preparations. (TIF) [file pone.0171366.s001.tif]

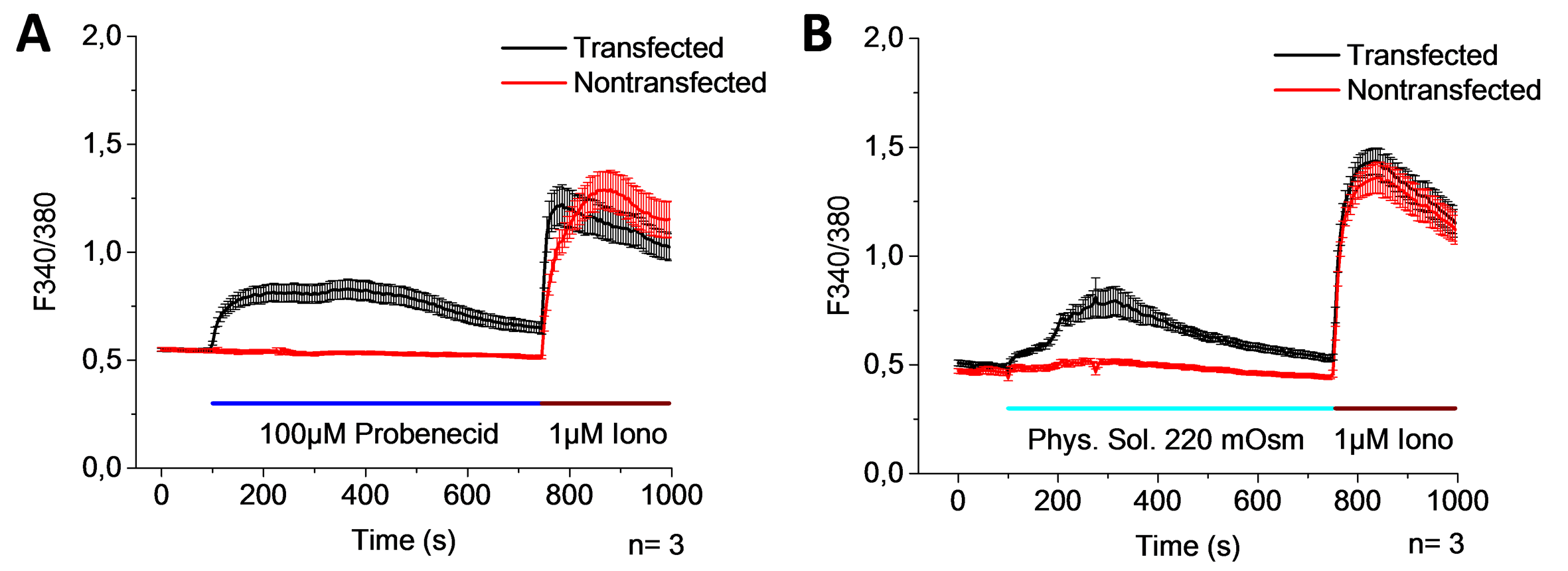

Supplement: S2 Fig — Time course of [Ca2+]i changes triggered by application of 100 μM of Probenecid (A) and 220 mOsm/kg hypotonic solution (B) in HEK293 cells that were transfected with a mTRPV2–IRES-GFP plasmid (black traces). TRPV2 expression was proven by the presence of the green fluorescence protein signal. As negative control, non-tranfected HEK293 cells were used (red traces). Ionomycin 1μM was used as a positive control. Graphs represent the mean ± SEM of 10 cells in each case. (TIF) [file pone.0171366.s002.tif]

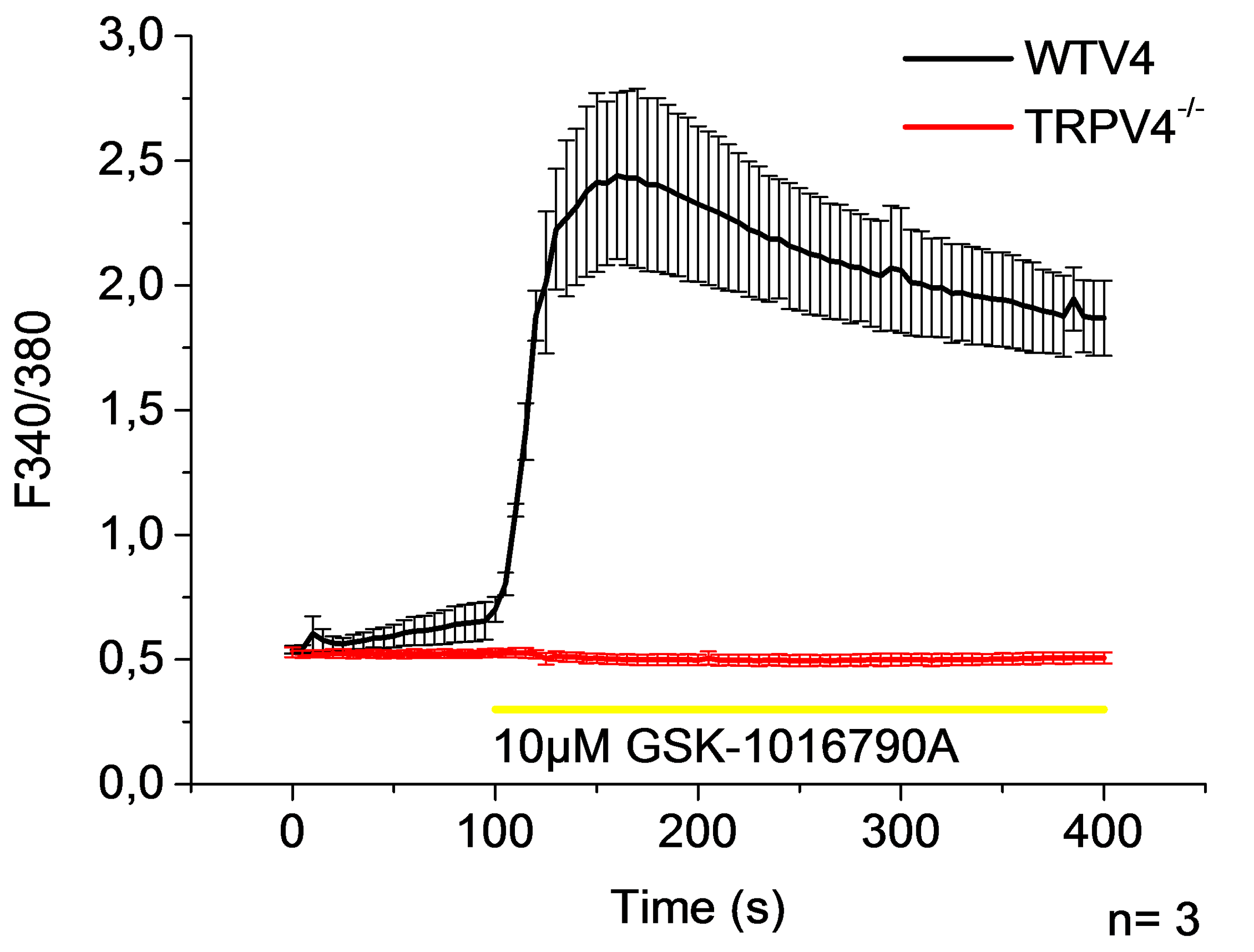

Supplement: S3 Fig — Time course of [Ca2+]i changes in Cardiac fibroblasts (CF) stimulated with 10 μM GSK-1016790A. CF were isolated from WT (black) and TRPV4 deficient (red) mice using retro-Langendorff-perfusion as previously described [40]. Graphs represent the mean ±SEM of 3 independent preparations. (TIF) [file pone.0171366.s003.tif]
